# Retraction: Elevated eukaryotic elongation factor 2 expression is involved in proliferation and invasion of lung squamous cell carcinoma

**DOI:** 10.18632/oncotarget.28803

**Published:** 2025-12-31

**Authors:** Yang Song, Bing Sun, LiHong Hao, Jun Hu, Sha Du, Xin Zhou, LiYuan Zhang, Lu Liu, LinLin Gong, XinMing Chi, Qiang Liu, ShuJuan Shao

**Affiliations:** ^1^Department of Histology and Embryology, Dalian Medical University, Dalian, China; ^2^Department of Chest Surgery, The First Affiliated Hospital of Dalian Medical University, Dalian, China; ^3^Institute of Cancer Stem Cell, Dalian Medical University Cancer Center, Liaoning, China; ^4^Liaoning Key Laboratory of Proteomics, Dalian Medical University, Liaoning, China; ^*^These authors have contributed equally to this work

**This article has been retracted:** Oncotarget has completed its investigation of this paper. Image forensic analysis confirmed that Figures 4C, 5A, 5C contain overlapping images. Figure 2B also has a splicing. In addition, Figure 1A overlaps with Figure 1A of reference [1]. The authors agreed with the errors and provided the data for correction. However, given the extent and number of required corrections, which exceeded a reasonable limit, the Editorial decision was made to retract the publication.The National Natural Science Foundation of China Supervision Committee also confirmed misconduct and concluded that the first author Song Yang, the corresponding author Shao Shujuan and others are responsible for the above issues. The authors have been notified about the decision.

Original article: Oncotarget. 2016; 7:58470–58482. 58470-58482. https://doi.org/10.18632/oncotarget.11298
